# Health literacy levels and predictors among type 2 diabetes patients in Qatar: An analytical cross-sectional study

**DOI:** 10.5339/qmj.2025.104

**Published:** 2025-12-04

**Authors:** Mohamad Alchawa, Rana Alasaad, Jihene Maatoug Maaloul, Mahmoud Zirie, Iheb Bougmiza

**Affiliations:** 1 Preventive Health, Primary Health Care Corporation, Doha, Qatar; 2 Internal Medicine Department, Endocrinology, West Bay Medicare, Doha, Qatar; 3 Community Medicine Department, Primary Health Care Corporation, Doha, Qatar; 4 Faculty of Medicine, Sousse University, Sousse, Tunisia; 5 Department of Endocrinology, Hamad Medical Corporation, Doha, Qatar *Email: malchawa@phcc.gov.qa

**Keywords:** health literacy, type 2 diabetes, HLS-EU-Q16, Qatar

## Abstract

**Background:**

Type 2 diabetes mellitus (T2DM) requires significant patient involvement, with health literacy playing a crucial role in patients’ ability to navigate their care. Qatar has one of the highest T2DM prevalence rates globally, yet research on health literacy in this population remains limited. The purpose of this study was to evaluate health literacy levels and predictors among Type 2 diabetes patients in Qatar.

**Methods:**

An analytical cross-sectional study was conducted, targeting patients diagnosed with type 2 diabetes. A total of 450 patients were randomly sampled, and data were collected through structured interviews using the European Health Literacy Survey Questionnaire - short version (HLS-EU-Q16) to measure health literacy. In addition to determining the prevalence of different health literacy levels, associations between health literacy and patient characteristics were examined using bivariate analysis. A regression model was employed to identify independent predictors of health literacy.

**Results:**

Of the 450 participants, 57.8% were male with a mean age of 51.6 years. 62.4 % demonstrated sufficient health literacy, 31.8 % problematic, and 5.8 % inadequate levels. Health literacy was significantly associated with participants’ age, education, occupation, income, living situation, diabetes duration, treatment, and complications (*P* < 0.05). Multivariable analysis showed that primary (adjusted odds ratio (AOR), 0.080; *P* < 0.001), no formal (AOR, 0.162; *P* = 0.008) and secondary education (AOR, 0.266; *P* = 0.001) each reduced the odds of higher literacy versus university education, while living with family (AOR, 2.843; *P* = 0.030) and being managed with oral medications alone (AOR, 3.230; *P* = 0.004) or no medication (AOR, 11.196; *P* = 0.038) increased the odds.

**Conclusion:**

Although a high proportion of patients had sufficient health literacy, many still struggled with problematic or inadequate levels, especially those with lower education or complex insulin regimens. Routine health literacy assessment and targeted, culturally appropriate education for high-risk groups should be embedded in diabetes services and national strategies.

## 1. INTRODUCTION

Type 2 diabetes mellitus (T2DM) is a multifaceted, chronic non-communicable disease (NCD) with a high burden of morbidity and mortality.^1^ It represents a significant global health challenge as one of the most prevalent NCDs. Recent data show that the number of adults living with diabetes has more than quadrupled over the past three decades, reaching over 800 million in 2022. During the same period, the global prevalence increased from 7% in 1990 to 14% in 2022.^2^ The socio-economic implications of this growing disease burden are vast. Healthcare expenditure for T2DM management and its complications was projected to reach $958 billion by 2045.^3^ The Middle East and North Africa (MENA) region is disproportionately affected by T2DM, exhibiting the highest regional prevalence globally. In 2024, 84.7 million adults aged 20 to 79 years in MENA were living with diabetes, a figure projected to reach 162.6 million by 2045.^4^ Qatar, one of the MENA region’s nations, has one of the highest prevalence rates of T2DM worldwide. A deterministic, age- and risk-factor–stratified model calibrated to national data predicts that annual new T2DM cases among Qatari nationals aged 15 to 64 years will almost double, rising from 978 in 2012 to 1907 by 2050.^5^ Epidemiological data indicate that approximately 20% of the Qatari population is affected by either prediabetes or diabetes mellitus. Al-Thani et al.,^6^ underscoring the urgent need for effective interventions.

Health literacy (HL), which the World Health Organization (WHO) defines as &quot;the cognitive and social skills which determine the motivation and ability of individuals to gain access to, understand, and use information in ways that promote and maintain good health,^7^&quot; is a crucial part of diabetes self-care. HL is consistently associated with the behaviors that underpin effective T2DM care. Patients with adequate HL are more likely to engage in recommended self-management practices such as daily foot care, blood glucose monitoring, healthy eating, and regular physical activity than those with limited HL.^8–10^ Systematic reviews also demonstrate a positive, though sometimes attenuated, association between HL and medication adherence, especially in ethnically diverse or low-income populations.^11^

The relationship between HL and T2DM outcomes is complex. While better HL often correlates with increased disease knowledge, its link to glycemic control is unclear.^12^ For example, some studies have reported no significant direct association between patients’ literacy levels and their glycemic outcomes once other variables are accounted for.^10,13^ Thus, HL might be viewed as one factor among many (e.g., healthcare system support and psychological well-being) that influence diabetes management success rather than as a sole determinant of outcomes. Factors like gender, age, ethnicity, income, disease duration, and education impact HL in T2DM patients.^14^ In the literature, HL is typically assessed using either generic or condition-specific tools, with the majority of studies opting for the former.^15,16^

Despite Qatar’s significant T2DM burden, research into HL and factors influencing it among T2DM patients remains limited. To our knowledge, no large-scale study has quantified HL levels or determinants among Qatar’s T2DM population, and only indirect evidence is available from the general population. For instance, a recent national survey found that almost half of Qatari adults have at least adequate HL.^17^ Accordingly, the present study investigates HL levels and their socio-demographic and clinical predictors among adults with T2DM. By identifying demographic and clinical predictors of inadequate HL in this multicultural setting, our study will pinpoint sub-groups that require customized support, inform the design of culturally and linguistically adapted interventions, and provide locally relevant benchmarks for monitoring progress toward national and regional HL targets. Ultimately, these insights can guide health-system planners in allocating resources toward interventions most likely to improve glycemic control and reduce complication-related expenditures.

## 2. METHODS

### 2.1 Study design, setting, and participants

An analytical cross-sectional study design was implemented in this study. The study was conducted in Qatar, a country with a population of 2.982 million as of February 2023.^18^ Participants were randomly sampled from all adult patients diagnosed with T2DM and registered in the major public healthcare system, unified under the electronic health record system managed by the Health Information and Communications Technology (HICT) department at Hamad Medical Corporation (HMC). The target population consisted of adult patients (aged 18 and above) diagnosed with T2DM (International Classification of Diseases, 10th Revision [ICD-10], codes E11.0 to E11.9) for 6 months or more.^19^ Exclusions included patients who could not understand or speak English or Arabic, pregnant females at the time of recruiting, and those unable or unwilling to give informed consent.

### 2.2 Sample size, sampling method, and recruitment procedures

Assuming a prevalence of 50% of sufficient HL levels and a 95% level of confidence (CI) with an error rate of 5%, the minimum required sample size was 384 participants based on OpenEpi® software version 3.01,^20^ using the standard formula for estimating a single population proportion with a large population assumption: *n* = Z^2^ × *p* × (1 – p)/d^2^, where the estimated prevalence (p) was 50%, the confidence level was 95%, and the margin of error was 5%. However, considering the power of the regression model assessing the relationship between independent variables and the outcome, a sample of 450 was deemed adequate.^21,22^ To account for the non-response, a total of 1000 T2DM patients were randomly sampled from the list of all adult T2DM patients registered in the unified health record system, and structured telephone interviews were conducted until 450 complete responses were obtained. The sampling was performed using a computer random number generator, and only completed questionnaires were counted toward the 450 required sample size.

Potential eligible participants were contacted by telephone. The study subjects were informed about the objective and structure of the study and queried about their consent to participate. Those who provided consent were then asked a screening question to exclude those recently diagnosed with diabetes (less than 6 months ago). Those who consented and were eligible enrolled in the study and were interviewed during the same telephone call.

### 2.3 Data collection and study variables

Data were collected through structured telephone interviews conducted by trained interviewers, using either Arabic or English versions of the questionnaire based on participant preference. The interviewers were trained before starting the data collection, and the questionnaire included items related to the following study variables.

**2.3.1 Independent variables**

The independent variables included demographic, individual, and clinical-related factors. Demographic and individual factors covered age, gender, nationality, and education based on Qatar’s educational categories,^23^ monthly household income in Qatari Riyal (QAR) [1QAR ≈ 0.27 US$], family size, marital status, employment status, occupation, and living condition.

Clinical characteristics included duration since diabetes diagnosis, medical treatment received, presence of diabetes complications, and other chronic diseases. Participants’ body mass index (BMI) was calculated from self-reported height and weight and categorized by WHO standards.^24^ Data about tobacco use status and whether participants had received formal diabetes education from a healthcare professional were also collected.

**2.3.2 Dependent variable**

The dependent variable was HL, measured by the European Health Literacy Survey Questionnaire - short version (HLS-EU-Q16).^25,26^ HL scores ranged from 0 to 16, with higher scores indicating higher levels of HL. Furthermore, the HLS-EU-Q16 score was categorized into three levels: inadequate HL (scores 0–8), problematic HL (scores 9–12), and sufficient HL (scores 13–16).^25,27^ The HLS-EU-Q16 is a concise and valid instrument, showing a strong correlation with its longer counterpart, HLS-EU-Q47, with a correlation coefficient of 0.82.^27^ It also aligns well with other HL evaluation tools like The Newest Vital Sign (NVS).^27^ It has been validated and used in many languages, including English and Arabic.^28,29^

### 2.4 Data analysis

The database was entered and analyzed using the Statistical Package for the Social Sciences (SPSS)^TM^ software (version 26.0. Armonk, NY: IBM Corp.; www.ibm.com/analytics/spss-statistics-software). Descriptive analysis was performed for the characteristics of the participants, and data were presented as frequencies and percentages for categorical variables. For continuous variables, normality was assessed using visual inspection of histograms and the Shapiro–Wilk test. and presented as mean and standard deviation (SD) for normally distributed variables, while median and interquartile range (IQR) were used for non-normally distributed variables. To determine the 95% confidence interval for the percentages of participants in each HL category based on their HLS-EU-Q16 scores, we employed the Wald method (https://measuringu.com/calculators/wald/) for a one-sample prevalence confidence interval.

Associations were assessed using bivariate analyses using the chi-square test or Fisher’s exact test for categorical variables. For continuous variables, and based on the normality assessment, the t-test, analysis of variance (ANOVA), Mann-Whitney U test, or Kruskal-Wallis test were used as appropriate. HL was categorized into three categories based on the developer’s recommendations.^25,27^ The internal reliability of the HLS-EU-Q16 was evaluated by computing Cronbach’s alpha coefficient. Ordinal regression was used to identify the independent predictors of HL and to calculate the adjusted odds ratios (AORs) with the 95% CIs. P-values < 0.05 (two-tailed) were considered statistically significant.

## 3. RESULTS

### 3.1 Sample realization and participants’ characteristics

Data collection was conducted from August 2022 to November 2022 and continued until a total of 450 complete responses were obtained. Out of the 1000 T2DM patients randomly sampled from the list of all adult T2DM patients registered in the unified health record system, we had to approach 991 eligible patients to achieve our sample of 450 complete responses. Out of the 991 participants approached, 398 were unreachable (unanswered calls, disconnected numbers, or wrong numbers), and 121 declined participation. Twenty-two interviews were terminated before completion and excluded. The remaining 450 fully completed interviews constitute the analytic sample, yielding a complete response rate of 45.4 %.

Out of the 450 participants included in the analysis, 260 (57.8%) were male, and 190 (42.2%) were female. The age of participants ranged from 20 to 83 years, with a mean (±SD) of 51.6 ± 10.6. The sample included participants from 32 different nationalities, with the most frequent one being the Qatari nationality (*n* = 122; 27.1%). Other predominant nationalities included Indian (*n* = 78; 17.3%), Egyptian (*n* = 42; 9.3%), Filipino (*n* = 39; 8.7%), and Sudanese (*n* = 35; 7.8%). In terms of education, 230 (51.1%) had completed university or higher education, while the numbers of those who had only primary school level or no formal education level were 55 (12.2%) and 39 (8.7%), respectively. Out of the 450 participants, 90 (20.0%) reported having a total monthly household income of less than 5000 QAR (1 QAR ≈ 0.27 US$), and 109 (24.2%) participants reported having a household income of more than 20,000 QAR.

As for marital status, the majority of the participants (*n* = 393; 87.3%) were married. However, only 362 (80.4%) were living with their families. The household family size ranged between 2 and 14 family members, with a mean of 5.2 ± 2.0. The employed individuals represented 64.4% (*n* = 290) of the recruited sample. Of those who were employed, the highest distribution of the occupation was among professionals (*n* = 181; 62.4%), followed by manual and craft workers (*n* = 73; 25.2%).

Participants had T2DM for an average of 8.73 years (± 7.18), ranging from 6 months to 37 years. For the current pharmacological treatment, 70.2% (*n* = 316) were using oral glucose-lowering medications, 6% (*n* = 27) used insulin only, and 20.2% (*n* = 91) used a combination of insulin and oral glucose-lowering medications. In addition, 3.6% (*n* = 16) of the participants were managing the disease with diet and exercise only, without any medications. Chronic diabetes complications were present in 28.7% (*n* = 129) of the sample, with the most common being ocular complications (16.7%; *n* = 75), and 9.8% (*n* = 44) of the participants reported having two or more diabetes-related complications.

Moreover, 61.3% (*n* = 276) had at least one other chronic disease, primarily hypertension (40.7%; *n* = 183) and dyslipidemia (32.2%; *n* = 145). The average BMI was 29.85 kg/m^2^ (±6.18), with the majority (76.7%; *n* = 345) being overweight or obese. Only 22.2% (*n* = 100) of the participants had a normal BMI (18.5–24.9 kg/m^2^). While most participants (76.9%; *n* = 346) had never used tobacco, 14.9% (*n* = 67) were current users and 8.2% (*n* = 37) were previous users. Lastly, a significant portion of T2DM patients (70.2%; *n* = 316) recalled receiving diabetes education sessions. Table 1 describes the participants’ sociodemographic and clinical characteristics.

### 3.2 HL levels

Utilizing the HLS-EU-Q16 questionnaire, the median HL score was 13 (IQR, 12–15), within the range indicating sufficient HL. Scores ranged from 1 to 16. When classified by the HLS-EU-Q16 recommended categories, most participants (*n* = 281; 62.4% [95% CI, 57.8%–66.8%]) demonstrated sufficient HL. However, a considerable portion showed problematic (*n* = 143; 31.8% [95%CI, 27.5%–36.2%]) or inadequate (*n* = 26; 5.8% [95% CI, 3.9%–8.3%]) HL.

**Figure 1:**
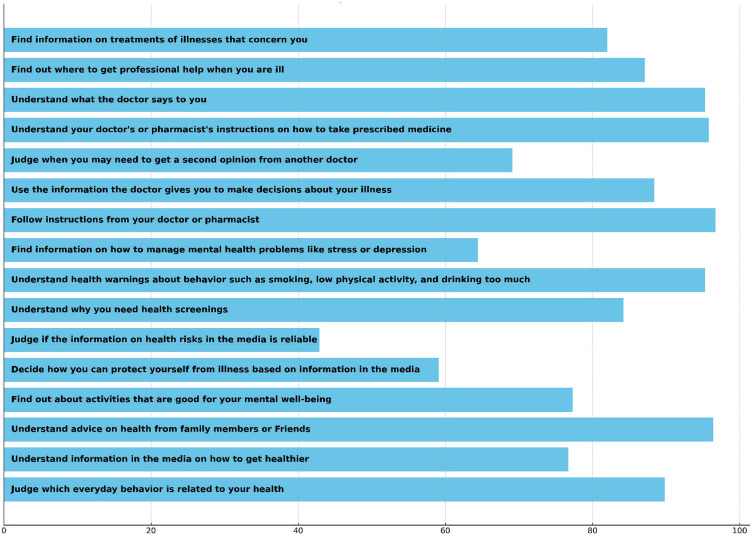
Distribution of participant responses (*n* = 450) to each item of the HLS-EU-Q16 health literacy questionnaire. Values represent the percentage of respondents who rated each item as “Easy” or “Very easy.”

In terms of HLS-EU-Q16 individual items responses, 108 participants (24.0%) responded “easy” to all questionnaire items. Most of the participants (*n* = 435; 96.7%) stated that they found following their doctors’ or pharmacists’ instructions fairly or very easy (the seventh question in the HLS-EU-Q16). In contrast, more than half of the participants (*n* = 257; 57.1%) found it fairly or very difficult to judge if the information on health risks in the media is reliable (the eleventh question in the HLS-EU-Q16). Figure 1 displays the percentages of participants’ responses for HLS-EU-Q16 items after recoding the answers into two categories: “difficult” and “easy.” The HLS-EU-Q16 had a Cronbach’s alpha of 0.85 in our study, denoting good internal consistency.

### 3.3 Associations between health literacy and participants’ characteristics

Several patient characteristics were found to be significantly associated with HL levels (Table 2). Older age was associated with lower HL, with only 36.4% of those over 70 having sufficient HL compared to 57.1% and 80.6% of those aged 30 years or less and those aged 31 to 40 years, respectively (*P* = 0.01). Higher education level was also associated with higher HL (*P* < 0.001), with university graduates being more likely to have sufficient HL. Professional workers had higher HL levels than manual/craft workers, with 74.2% (*n* = 161) of the professional workers having a sufficient HL level, while only 37.0% (*n* = 27) of the manual and craft workers had the same level of HL (*P* < 0.001). A significant association was observed between monthly household income and HL (*P* = 0.001). Sufficient HL was lowest in the lowest income group (<5000 QAR; 45.6%), and highest among those with middle incomes (5000–20,000 QAR; ranging from 69.1% to 73.9%). Notably, the highest income group (>20,000 QAR) had a lower proportion with sufficient HL (55.0%) than the middle-income categories. Living situation was significantly associated with HL (*P* < 0.001). Sufficient HL was found in 66.3% of those living with family and 64.4% of those living alone. In contrast, only 27.9% of participants sharing accommodation with friends or co-workers had sufficient HL.

Shorter diabetes duration was associated with higher HL, as 71.3% of those with relatively recent disease diagnosis (≤3 years) had sufficient HL, while only 53.5% of those with ≥ 10 years of disease duration had sufficient HL (*P* = 0.031). The treatment being received was significantly associated with HL levels (*P* < 0.001). Sufficient HL was observed in 68.0% of those on oral medications only and in 93.8% of those not taking any diabetes medications. In comparison, only 55.6% of those on insulin alone and 39.6% of those on both oral medications and insulin had sufficient HL. Additionally, the presence of diabetes complications was also associated with lower HL (*P* < 0.001): only 44.2% of participants with at least one diabetes complication had sufficient HL, compared to 69.8% of those without complications. The presence of other chronic conditions besides diabetes was also associated with lower HL (*P* = 0.008). Participants with other chronic diseases besides diabetes were less likely to have sufficient HL (57.2%) compared to those without any comorbidities (70.7%). No associations were found between HL and gender, marital status, employment, BMI, prior DM-related education, or tobacco use.

### 3.4 Predictors of HL levels among T2DM patients

Using the ordinal logistic regression analysis, the model revealed that the predictors in the model significantly improved the explanation of HL levels compared to a null model. The pseudo-R^2^ measures suggested that 37.7% (Nagelkerke’s R^2^) of the variability in HL levels was accounted for by the predictors in the model.

The analysis identified education level, living arrangement, and diabetes medication type as significant predictors of HL levels. Participants without formal education had significantly lower odds of higher HL than those with university or higher education (AOR = 0.162 [95% CI, 0.042–0.629], *P* = 0.008). The odds were even lower for participants with primary education (AOR = 0.080 [95% CI, 0.024–0.263]; *P* < 0.001). Those with secondary education also had lower odds compared to those with university or higher levels of education (AOR = 0.266 [95% CI, 0.122–0.576]; *P* = 0.001).

Participants living with family had higher odds of sufficient HL compared to those in shared accommodation (with friends or co-workers; AO*R* = 2.843 [95% CI, 1.104–7.325]; *P* = 0.030). Moreover, participants who were not on diabetes medications had significantly higher odds of better HL levels compared to those on both oral medications and insulin (OR = 11.196 [95% CI, 1.138–110.201]; *P* = 0.038). Participants on oral medications also had significantly higher odds compared to those on both oral medications and insulin (OR = 3.230 [95% CI, 1.447–7.207]; *P* = 0.004). Other variables, including age, income, DM duration, occupation, DM complications, and the presence of other chronic diseases, were not significant predictors (Table 3).

The model’s goodness of fit was also confirmed by the Pearson (χ^2^ = 477.893; *P* = 0.067) and Deviance (χ^2^ = 286.328; *P* = 1.000) tests.

## 4. DISCUSSION

Diabetes mellitus, especially Type 2 (T2DM), is a global health issue, now considered a pandemic.^30^ Qatar has a notably higher T2DM prevalence than many regions,^4^ highlighting the need to understand its management and outcomes there. This study evaluates HL levels in Qatar’s T2DM patients and potential influencing predictors. Our sample of 450 adult T2DM patients provides a representative view of Qatar’s T2DM community. The findings from this study offer valuable insights into the HL landscape among T2DM patients in the country.

Our findings revealed that nearly two-thirds (62.4%) of type 2 diabetes patients in Qatar demonstrated sufficient health literacy, with the remaining participants experiencing either problematic (31.8%) or inadequate (5.8%) levels. These results present a more favorable health literacy profile compared to several international studies. A systematic review by Abdullah et al. reported that limited health literacy among type 2 diabetes patients ranged from 7.3% to 76.3% across various populations, with a pooled global prevalence of 32.5%.^31,32^ The relatively high proportion of sufficient health literacy in our sample may reflect Qatar’s high Human Development Index and substantial investments in healthcare infrastructure and education.

Comparatively, our participants demonstrated higher levels of health literacy than those reported by Schillinger et al.,^33^ in which 38% of T2DM patients exhibited inadequate HL. While such cross-study comparisons provide useful context, they should be interpreted with caution due to differences in measurement tools, healthcare systems, and population characteristics. Similarly, our findings were also more favorable than those of Mohammadi et al.,^34^ where lower HL levels were observed. This discrepancy may reflect the higher educational attainment within our sample, as well as differences in access to healthcare and the availability of structured diabetes education in Qatar. However, other unmeasured factors, such as cultural perceptions of health information or the effectiveness of national health programs, could also contribute.

While there is a scarcity of studies assessing HL among diabetes patients in Qatar, research from neighboring Saudi Arabia offers some comparative insights. One study reported that 68.7% of T2DM patients had adequate HL levels,^35^ slightly higher than ours at 62.4%. Another more recent study from Saudi Arabia in 2022 reported only 37.1% of T2DM patients with adequate HL.^36^ This discrepancy could be attributed to differences in sample sizes, the educational backgrounds of participants, and the tools used for assessment.

Our study utilized a general health literacy instrument and included a diverse, relatively well-educated patient population, which may partly explain why our literacy levels trend higher than some reports from more heterogeneous or less educated populations in the region. It is also worth noting that health literacy measurement lacks standardization across studies; some use general instruments, whereas others employ diabetes-specific literacy tools, which can yield different estimates.

Our analysis revealed that patients with inadequate HL were more likely to exhibit certain demographic and clinical characteristics, such as older age, lower education, and diabetes complications, among others. However, upon regression analysis, only higher education, diabetes treatment type, and living arrangements remained significantly associated with HL. This aligns with findings from other studies in various regions, which have consistently shown a strong correlation between education and HL levels.^33,34,36^ These findings reinforce that improving patient education (both in the general and health-specific sense) could directly translate to higher health literacy levels.

In contrast, age did not remain a significant independent predictor of literacy in our adjusted model, even though bivariate analysis suggested older patients tend to have lower literacy. This result diverges slightly from some studies (e.g., Schillinger et al.) that identified older age as associated with poorer literacy.^33^ The attenuation of the age effect in our multivariable model likely indicates that age was confounded by factors such as duration of diabetes and the complexity of treatment regimens, which often increase with age. In other words, once we controlled variables like disease duration and treatment type, age itself no longer predicted health literacy. This phenomenon, where age loses its significance in relation to HL when adjusted for other variables, has been observed in other studies, such as Pavão et al.’s research in Brazil.^37^ This suggests that it may not be chronological age per se, but rather factors correlated with age, like long-standing diabetes or disease complications, that influence literacy levels.

Likewise, variables such as income and the presence of diabetes complications, which showed associations with HL in bivariate analysis, did not retain significance in the adjusted model. This likely reflects the confounding influence of education and treatment complexity, respectively. Higher income often coincides with higher education (which was included in the model), and patients with complications typically have longer disease duration or intensive treatment, factors already accounted for.

One notable finding from our study was the lack of a significant difference in HL levels between participants who had received diabetes-related education and those who hadn’t. This can be attributed to our focus on general HL rather than diabetes-specific HL. Diabetes education sessions typically concentrate on disease-specific topics, which might not significantly impact general HL. The same rationale applies to the lack of association found between disease duration and HL level, as we assessed general HL rather than diabetes-specific HL, resulting in no correlation with disease duration. In our study, the lack of a measurable difference in general literacy between those who did and did not receive diabetes education suggests that such training, while valuable for disease management, does not necessarily elevate patients’ broader health navigation skills or literacy outside the diabetes domain. This interpretation is supported by evidence from other settings, for instance, a study in Ethiopia by Mogessie et al.,^38^ used a diabetes-specific health literacy questionnaire and found that patients with longer disease duration and those who had received diabetes education had significantly better diabetes-related literacy. Those associations were absent from our findings, probably because our instrument captured general health literacy. These insights highlight an important practical point that interventions aiming to improve health literacy should be tailored to the type of literacy being targeted to improve (generally versus disease-specific), and researchers should choose measurement tools accordingly. Our results suggest that while conventional diabetes education improves disease management competencies, additional strategies might be needed to boost patients’ general health literacy, such as training in how to seek, interpret, and use health information beyond the immediate scope of diabetes self-care.

The present findings have significant implications for public health practice and policy in Qatar and the broader region. The fact that over one-third of T2DM patients in our study struggle with health literacy signals a clear need for strengthened interventions to support patient understanding and engagement. Qatar’s healthcare authorities have acknowledged the significance of educating and empowering patients in managing chronic diseases. However, the response is still inadequate compared to the growing epidemic, and most prevention and health promotion efforts remain limited in scope, generic, and didactic.^39^ Recently, Qatar has developed the Action Plan 2024–2030 on Obesity, Diabetes, and Modifiable Risk Factors, which includes 58 projects.^40^ One of the main areas under this action plan is public empowerment. This action plan integrates ongoing initiatives such as screenings and primary care empowerment with new projects. However, more structured programs are needed, especially in the primary healthcare settings in Qatar, that are culturally tailored to target diabetes patients’ health literacy.

This present study has several strengths, as it stands as one of the first studies in the country to offer a comprehensive assessment of HL levels among T2DM patients and investigate its predictors. The generalizability of our findings is enhanced by deriving results from a random subset of the majority of T2DM patients in Qatar and is potentially applicable to other Gulf Cooperation Council (GCC) countries with similar demographics. The diversity of our sample, with participants of 32 nationalities and minimal exclusion criteria, combined with the use of validated tools, ensures the external validity and reliability of our findings.

However, this study comes with a few limitations. Despite rigorous interviewer training, the telephone-only recruitment strategy may have excluded patients without reliable phone access or those uncomfortable with phone interviews, groups that often include older adults or individuals with very limited literacy, potentially inflating observed literacy levels. The reliance on self-reported data could lead to potential social desirability biases, especially concerning weight and HL questions. The study’s cross-sectional design limits the establishment of temporality between variables. Additionally, the exclusive use of Arabic and English questionnaires might exclude certain linguistic groups in Qatar.

## 5. CONCLUSIONS AND RECOMMENDATIONS

In conclusion, this study highlights clear gaps in health literacy among T2DM patients in Qatar, particularly among those with lower educational attainment, living in shared accommodation, or on complex medication regimens. These findings should prompt healthcare providers to systematically screen for health literacy during patient intake and follow-up, using validated tools. Identified at-risk patients should be referred to tailored, language- and literacy-appropriate diabetes education programs, which can be developed collaboratively by health educators, physicians, patient representatives, and diabetes nurse specialists. These interventions should incorporate practical tools such as materials with simple language, large fonts, and pictograms. Clinicians should be trained to use evidence-based communication techniques like the &apos;teach-back&apos; method to confirm patient understanding during consultations.

At the policy level, our data support the integration of health literacy objectives and assessment metrics into national diabetes strategies and quality improvement initiatives, with dedicated resources for training healthcare providers in effective communication and health education. Additionally, it might be beneficial to embed plain language standards into all patient-facing documents and develop digital health tools with user-friendly interfaces designed for diverse literacy levels. Healthcare administrators can use these results to justify investment in health literacy interventions and to develop key performance indicators for monitoring progress in HL improvement across facilities.

Finally, these findings provide a strong rationale for future intervention studies to evaluate the impact of targeted HL programs on self-management and clinical outcomes in high-risk groups.

## ETHICAL STATEMENT

The study protocol was approved by the institutional review board of Hamad Medical Corporation under protocol ID (MRC-01-22-139). Informed consent was acquired from all participants. The study was conducted in full conformance with the principles of the “Declaration of Helsinki” and Good Clinical Practice.

## AVAILABILITY OF DATA

The datasets generated during and/or analyzed during the current study are available from the corresponding author on reasonable request.

## DECLARATION OF INTEREST

The authors have no competing interests to declare.

## Figures and Tables

**Table 1.** Basic sociodemographic and clinical characteristics of the study participants (*n* = 450).

Characteristic	*n*	%
**Gender**		
Male	260	57.8
Female	190	42.2
**Age, years**		
≤30	7	1.6
31–40	67	14.9
41–50	124	27.6
51–60	156	34.7
61–70	85	18.9
≥71	11	2.4
**Nationality**		
Qatari	122	27.1
Non-Qatari	328	72.9
**Educational level**		
No formal education	39	8.7
Primary	55	12.2
Secondary	126	28.0
University or higher	230	51.1
**Marital status**		
Married	393	87.3
Single	57	12.7
**Employment status**		
Employed	290	64.4
Not employed	111	24.7
Retired	49	10.9
**Occupation (*n* = 290 employed participants)**		
Professional	181	62.4
Manual and craft worker	73	25.2
Business person	22	7.6
Military/police person	14	4.8
**Monthly household income^a^**		
<5000 QAR	90	20.0
5000–10,000 QAR	111	24.7
10,001–15,000 QAR	81	18.0
15,001–20,000 QAR	59	13.1
>20,000 QAR	109	24.2
**Living situation**		
Alone	45	10.0
With family	362	80.4
Sharing accommodation with friends	16	3.6
Sharing accommodation with co-workers	27	6.0
**Family size (*n* = 362 living with family)**		
2–3 members	62	17.1
4–6 members	214	59.1
7–8 members	63	17.4
≥9 members	23	6.4
**Diabetes duration (years)**		
≤3	115	25.6
4–<10	178	39.6
≥10	157	34.9
**Diabetes treatment**		
Oral medications only	316	70.2
Insulin only	27	6.0
Both oral medications and insulin	91	20.2
No medications	16	3.6
**Diabetes complications^b^**		
Retinopathy	75	16.7
Diabetic kidney disease	32	7.1
Diabetic neuropathy	35	7.8
Cardiovascular disease	27	6.0
Foot ulcer/amputation	20	4.4
No diabetes complications	321	71.3
**Other chronic diseases^b^^,^^c^**		
Hypertension	183	40.7
Dyslipidemia	145	32.2
Heart diseases (non-diabetic)	33	7.3
Hypothyroidism	19	4.2
Hyperthyroidism	3	0.7
Cancer	8	1.8
**Chronic kidney disease (non-diabetic)**	7	1.6
Asthma	5	1.1
Other	24	5.3
No comorbidities	174	38.7
**BMI**		
Underweight	5	1.1
Normal	100	22.2
Overweight	148	32.9
Obese	197	43.8
**Tobacco use**		
Never	346	76.9
Current user	67	14.9
Previous user	37	8.2
**Previous diabetes education^d^**		
Yes	316	70.2
No	134	29.8

BMI: Body mass index; *n*: Frequency; %: Percentage.

a In QAR: Qatari Riyal (1QAR ≈ 0.27 US$).

b Percentages are out of 450 participants, and they are neither exhaustive nor mutually exclusive.

c Defined as a disease or condition that usually lasts for 3 months or longer.

d Defined as attending a structured diabetes self-management education session provided by a health care professional.

**Table 2.** The associations between health literacy categories and participants’ characteristics using bivariate analysis.

Characteristic	Health literacy			χ²; (*P*-value)
	Inadequate *n* (%)	Problematic *n* (%)	Sufficient *n* (%)	
**Gender**				
Male	17 (6.5)	78 (30.0)	165 (63.5)	1.33; (0.514)
Female	9 (4.7)	65 (34.2)	116 (61.1)	
**Age, years**				
≤30	1 (14.3)	2 (28.6)	4 (57.1)	21.67; (0.010)^*^
31–40	2 (3.0)	11 (16.4)	54 (80.6)	
41–50	5 (4.0)	48 (38.7)	71 (57.3)	
51–60	9 (5.8)	46 (29.5)	101 (64.7)	
61–70	7 (8.2)	31 (36.5)	47 (55.3)	
≥71	2 (18.2)	5 (45.5)	4 (36.4)	
**Ethnicity**				
Arab^a^	15 (5.4)	83 (29.7)	181 (64.9)	1.85 (0.397)
Non-Arab^b^	11 (6.4)	60 (35.1)	100 (58.5)	
**Educational level**				
No formal education	5 (12.8)	21 (53.8)	13 (33.3)	85.34 (<0.001)^*^
Primary	9 (16.4)	32 (58.2)	14 (25.5)	
Secondary	9 (7.1)	47 (37.3)	70 (55.6)	
University or higher	3 (1.3)	43 (18.7)	184 (80.0)	
**Marital status**				
Married	21 (5.3)	123 (31.3)	249 (63.4)	1.91 (0.371)^*^
Single	5 (8.8)	20 (35.1)	32 (56.1)	
**Current employment status**				
Employed	15 (5.2)	87 (30.0)	188 (64.8)	2.07 (0.355)
Not employed (including retired)	11 (6.9)	56 (35.0)	93 (58.1)	
**Occupation (*n* = 290 employed participants)**				
Professional^c^	6 (2.8)	50 (23.0)	161 (74.2)	33.80 (<0.001)^*^
Manual and craft worker	9 (12.3)	37 (50.7)	27 (37.0)	
**Monthly household income^d^**				
<5000 QAR	10 (11.1)	39 (43.3)	41 (45.6)	25.72 (0.001)^*^
5000–10,000 QAR	4 (3.6)	25 (22.5)	82 (73.9)	
10,001–15,000 QAR	3 (3.7)	22 (27.2)	56 (69.1)	
15,001–20,000 QAR	4 (6.8)	13 (22.0)	42 (71.2)	
>20,000 QAR	5 (4.6)	44 (40.4)	60 (55.0)	
**Living situation**				
Alone	3 (6.7)	13 (28.9)	29 (64.4)	27.55 (<0.001)^*^
With family	15 (4.1)	107 (29.6)	240 (66.3)	
Sharing accommodation with friends or co-workers	8 (18.6)	23 (53.5)	12 (27.9)	
**Family size (*n* = 362 living with family)**				
2–3 members	1 (1.6)	14 (22.6)	47 (75.8)	5.66 (0.456)^*^
4–6 members	9 (4.2)	70 (32.7)	135 (63.1)	
7–8 members	3 (4.8)	18 (28.6)	42 (66.7)	
≥9 members	2 (8.7)	5 (21.7)	16 (69.6)	
**Diabetes duration (years)**				
≤3	3 (2.6)	30 (26.1)	82 (71.3)	10.59 (0.031)
4–<10	11 (6.2)	52 (29.2)	115 (64.6)	
≥10	12 (7.6)	61 (38.9)	84 (53.5)	
**Diabetes treatment**				
Oral medications only	15 (4.7)	86 (27.2)	215 (68.0)	34.63 (<0.001)^*^
Insulin only	1 (3.7)	11 (40.7)	15 (55.6)	
Both oral medications and insulin	9 (9.9)	46 (50.5)	36 (39.6)	
No medications	1 (6.3)	0 (0.0)	15 (93.8)	
**Diabetes complications**				
At least one diabetes complication	15 (11.6)	57 (44.2)	57 (44.2)	29.12 (<0.001)
No diabetes complications	11 (3.4)	86 (26.8)	224 (69.8)	
**Other chronic diseases**				
At least one comorbidity	21 (7.6)	97 (35.1)	158 (57.2)	9.77 (0.008)
No comorbidities	5 (2.9)	46 (26.4)	123 (70.7)	
**BMI**				
Underweight	1 (20.0)	2 (40.0)	2 (40.0)	8.99 (0.153)^*^
Normal	4 (4.0)	34 (34.0)	62 (62.0)	
Overweight	6 (4.1)	39 (26.4)	103 (69.6)	
Obese	15 (7.6)	68 (34.5)	114 (57.9)	
**Tobacco use**				
Never	17 (4.9)	107 (30.9)	222 (64.2)	5.44 (0.228)^*^
Current user	4 (6.0)	25 (37.3)	38 (56.7)	
Previous user	5 (13.5)	11 (29.7)	21 (56.8)	
**Previous diabetes education**				
Yes	17 (5.4)	99 (31.3)	281 (62.4)	0.480 (0.787)
No	9 (6.7)	44 (32.8)	81 (60.4)	

a Includes Qatari, Egyptian, Sudanese, Jordanian, Syrian, Yemeni, Tunisian, Palestinian, Lebanese, Saudi Arabian, Algerian, Iraqi, Omani, Somali, Bahraini, and Moroccan.

b The majority of non-Arab participants were Indians (*n* = 78, 17.3%) and Filipinos (*n* = 39, 8.7%).

c Includes business people, military, and police.

d In QAR: Qatari Riyal (1QAR ≈ 0.27 US$).

*n*: Frequency; %: Percentage; χ^2^: Chi-square statistic; BMI: Body mass index;

* Fisher-exact test.

**Table 3.** Predictors of health literacy among type 2 diabetes patients as identified by ordinal regression analysis.

Variable		B	Std. Error	*P*-value	OR	95% Confidence interval for OR	
						Lower	Upper
Age	30 or less	−1.893	1.3958	0.175	0.151	0.010	2.322
	31–40	0.369	0.6441	0.566	1.447	0.409	5.113
	41–50	−0.495	0.5276	0.348	0.609	0.217	1.713
	51–60	−0.171	0.5287	0.746	0.843	0.299	2.375
	61 or more	0^a^	.	.	1	.	.
Education level	No formal education	−1.817	0.6903	0.008	0.162	0.042	0.629
	Primary	−2.526	0.6072	0.000	0.080	0.024	0.263
	Secondary	−1.326	0.3952	0.001	0.266	0.122	0.576
	University or higher	0^a^	.	.	1	.	.
Household income^*^	<5000	0.553	0.5913	0.350	1.738	0.545	5.539
	5000–10,000	0.496	0.4827	0.305	1.641	0.637	4.227
	10,001–15,000	0.447	0.5131	0.384	1.564	0.572	4.274
	15,001–20,000	0.858	0.5668	0.130	2.358	0.776	7.161
	>20,000	0^a^	.	.	1	.	.
Living condition	Alone	0.437	0.5304	0.410	1.548	0.548	4.378
	With family	1.045	0.4828	0.030	2.843	1.104	7.325
	Sharing accommodation (with friends or co-workers)	0^a^	.	.	1	.	.
Diabetes duration	3 years or less	−0.283	0.4590	0.538	0.754	0.307	1.853
	4 to less than 10 years	−0.451	0.3949	0.254	0.637	0.294	1.382
	10 years or more	0^a^	.	.	1	.	.
Diabetes treatment	No medications	2.416	1.1667	0.038	11.196	1.138	110.201
	Oral medications	1.172	0.4096	0.004	3.230	1.447	7.207
	Insulin	0.638	0.7467	0.393	1.892	0.438	8.176
	Both oral medications and insulin	0^a^	.	.	1	.	.
Occupation	Professional^†^	0.209	0.5455	0.702	1.232	0.423	3.590
	Manual and craft worker	0^a^	.	.	1	.	.
Presence of diabetes complications	No	0.564	0.3657	0.123	1.757	0.858	3.599
	Yes	0^a^	.	.	1	.	.
Presence of other chronic diseases	No	0.315	0.3038	0.300	1.370	0.755	2.485
	Yes	0^a^	.	.	1	.	.

B: Beta coefficient; OR: Odds ratio.

* In QAR: Qatari Riyal (1QAR ≈ 0.27 US$).

† Includes business people, military, and police.

a Set to zero because this parameter is redundant.
